# When amiodarone-induced thyroiditis meets cardiomyopathy with excessive trabeculation: a case report

**DOI:** 10.3389/fcvm.2023.1212965

**Published:** 2023-07-21

**Authors:** Dora Gašparini, Damir Raljević, Vesna Pehar-Pejčinović, Tihana Klarica Gembić, Viktor Peršić, Tamara Turk Wensveen

**Affiliations:** ^1^ Department of Histology and Embryology, Faculty of Medicine, University of Rijeka, Rijeka, Croatia; ^2^ Center for Diabetes, Endocrinology and Cardiometabolism, Hospital for Medical Rehabilitation of the Heart and Lung Diseases and Rheumatism Thalassotherapia Opatija, Opatija, Croatia; ^3^ Department of Rehabilitation and Sports Medicine, Faculty of Medicine, University of Rijeka, Rijeka, Croatia; ^4^ Division of Cardiology, Hospital for Medical Rehabilitation of the Heart and Lung Diseases and Rheumatism Thalassotherapia Opatija, Opatija, Croatia; ^5^ Department for Nuclear Medicine, Clinical Hospital Center Rijeka, Rijeka, Croatia; ^6^ Department of Internal Medicine, Faculty of Medicine, University of Rijeka, Rijeka, Croatia; ^7^ Department of Endocrinology, Diabetes and Metabolic Diseases, Clinic of Internal Medicine, Clinical Hospital Center Rijeka, Rijeka, Croatia

**Keywords:** amiodarone, case report, isolated noncompaction of the ventricular myocardium, prednisone, ventricular tachycardia

## Abstract

**Introduction:**

Amiodarone is a potent antiarrhythmic medication used to treat life-threatening ventricular arrhythmias; however, its well-established adverse effect is a thyroid disorder. Amiodarone-induced thyroiditis (AIT), a clinical entity involving two types with different etiopathology and treatment approaches, may occur at the beginning or even several years after amiodarone treatment discontinuation. The toxicity profile of amiodarone becomes especially important in young patients with lifelong cardiac disorders, which are often refractory to other antiarrhythmic drugs. Herein, we report the first case of non-sustained ventricular tachycardia (NSVT), an unusual presentation of type II AIT, in a young male patient who was previously diagnosed with left ventricular cardiomyopathy with excessive trabeculation.

**Case report:**

A 36-year-old male non-athlete presented with tiredness during regular follow-up. Continuous electrocardiographic monitoring (cECG) revealed NSVT, whereas echocardiography and cardiac magnetic resonance imaging detected discrete structural and functional changes that could not fully explain the observed cECG report. Conversely, an unmeasurably low thyrotropin level on admission and previous exposure to amiodarone pointed the diagnostic pathway in the direction of the thyroid gland. Elevated free thyroxine and undetectable autoantibody titers with unremarkable sonographic findings raised clinical suspicion of type II AIT. Scintigraphic imaging with ^99m^Tc-2-methoxyisobutylisonitrile (sestamibi) revealed decreased thyroid uptake; hence, prednisone was introduced for treatment. Clear improvements in both biochemical and electrocardiographic parameters were observed after immunomodulatory treatment of type II AIT in this young patient with cardiomyopathy and excessive trabeculation.

**Conclusion:**

Treatment of reversible causes of cardiac rhythm abnormalities such as type II AIT should be considered before choosing other treatment modalities, particularly in patients with structural cardiac disorders. The importance of a multidisciplinary approach in complex cases such as the one reported, thus, cannot be emphasized enough.

## 1. Introduction

Cardiovascular disease is the leading cause of death worldwide, and global strategies to reduce its incidence, morbidity, and mortality are continuously being developed (1). The insidious nature of cardiac conduction disease is a strong predictor of sudden cardiac death, but adequate medical therapy in combination with an implantable cardioverter defibrillator is the gold standard for risk reduction in patients with non-ischemic cardiac disease (2). Amiodarone is a potent class III antiarrhythmic medication used to treat life-threatening ventricular arrhythmias. However, a well-established adverse effect of amiodarone treatment is a thyroid disorder, occurring in up to 20% of patients receiving this type of treatment (3). Interestingly, amiodarone-induced thyroid disorders, including hyperthyroidism or hypothyroidism, may occur at the beginning of or even several years after amiodarone treatment discontinuation, both in patients with underlying thyroid pathology and those with a seemingly intact thyroid gland (3–5).

On the hyperthyroid part of the spectrum, amiodarone-induced changes are classically divided into type I or iodine-induced thyroiditis, and type II or destructive thyroiditis. Type I amiodarone-induced thyroiditis (AIT) occurs in patients with a prominent increase in thyroid hormone production, as a consequence of an underlying thyroid condition such as latent Graves' disease or multinodular goiter. Conversely, the direct cytotoxic effect of amiodarone in patients with no underlying thyroid disorder leads to the destructive phenotype of type II AIT with increased release of thyroid hormones. Although mixed types of AIT may be observed, the distinction between type I and type II is the crucial step in the management of these patients because of the difference in the therapeutic approach consisting of thyroid suppressant or corticosteroid medications, respectively (5). While decreased ^99m^Tc-sestamibi thyroid uptake on scintigraphy and absent vascularity on color flow Doppler ultrasound suggest the diagnosis of type II AIT, detectable radioiodine uptake, increased vascularity, and elevated serum levels of autoantibodies directed towards thyroid peroxidase, thyroglobulin and/or thyrotropin receptor are typically found in patients with type I AIT (5–7). Research has shown that the prevalence of type II AIT is increasing, which may be contributed to by the avoidance of amiodarone administration to patients with pre-existing thyroid disorders, aided by the availability of other effective antiarrhythmic agents (8).

Regardless of the type, AIT is associated with increased mortality independent of thyroid hormone levels and age but is strongly associated with severe left ventricular dysfunction (9). Few cases of AIT mimicking low cardiac output syndrome in patients with dilated non-ischemic cardiomyopathy have been reported so far (10–14). Nevertheless, the toxicity profile of amiodarone becomes especially important in young patients with lifelong structural cardiac disorders often refractory to other antiarrhythmic drugs (15). Hereby, we report the first case of non-sustained ventricular tachycardia (NSVT), an unusual presentation of type II amiodarone-induced thyroiditis, in a patient with the diagnosis of left ventricular cardiomyopathy with excessive trabeculation, a rare cardiac disorder.

## 2. Case description

**Figure 1:**
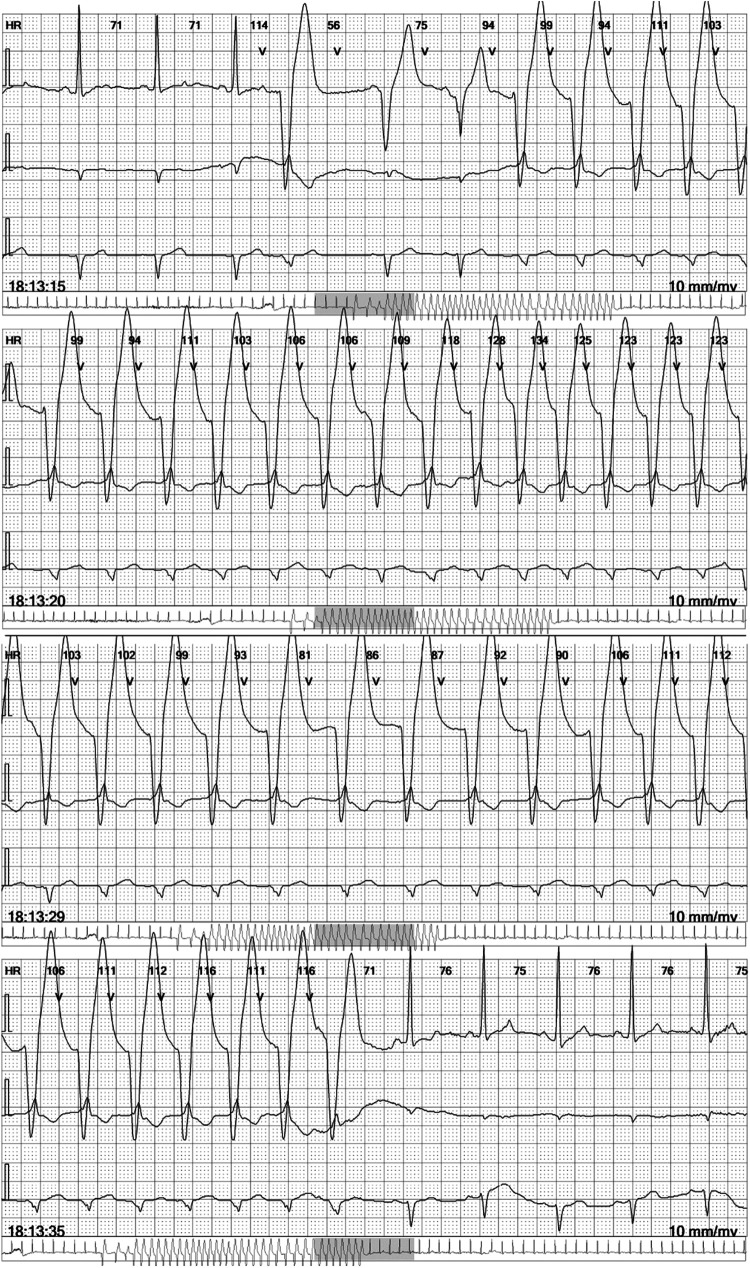
Non-sustained ventricular tachycardia in the continuous electrocardiographic monitoring report. Selected four electrocardiographic (ECG) strips at 25 mm/s, each showing three leads, as a part of the 6-lead ECG holter recorder DMS 300-4l 24 h report (DM Systems Co., Ltd., Beijing, China) after visualization with Holter Analyzer Software version 4.0076 (DM Systems Co., Ltd., Beijing, China).

A 36-year-old male non-athlete presented with tiredness and a slight limitation of physical activity during regular follow-up for a structural cardiac disorder. The diagnosis of cardiomyopathy with excessive trabeculation (16), or according to the old nomenclature of left ventricular non-compaction cardiomyopathy (LVNC) (17), was made two years before the current admission according to the morphological criteria valid at that time. The diagnosis was made after imaging studies were performed in this patient who presented with stabbing chest pain and a family history of sudden cardiac arrest in a second-degree relative. At the time of diagnosis, electrocardiographic assessment revealed a paroxysm of atrial fibrillation, for which he was prescribed carvedilol 3.125 µg once daily, acetylsalicylic acid 100 mg once daily, and amiodarone 200 mg. While the patient was adherent to carvedilol treatment, acetylsalicylic acid and amiodarone were discontinued after less than four months of treatment, more than a year before the current admission. At the time of current admission, no loss of consciousness, palpitations, or chest pain were reported. However, the patient confirmed increased sweating and weight loss as the main complaints, along with tiredness. Initial assessment of vital signs revealed a blood pressure of 120/80 mmHg and a pulse rate of 70 beats per min. The patient was afebrile and eupneic. Clinical examination results were otherwise unremarkable. The 24 h continuous electrocardiographic monitoring (cECG) revealed non-sustained ventricular tachycardia (Figure 1). The cECG showed ventricular heterotopy of 310 solitary and 80 pairs of ventricular extrasystoles, as well as 114 paroxysms of NSVT with a heart rate of up to 200 bpm and a duration of up to 20 s (Figure 1, Supplementary Table S1). Additionally, 7 solitary supraventricular extrasystoles and 11 paroxysms of ectopic atrial rhythm with a heart rate of up to 140 bpm and a duration of up to 10 min have been reported (Supplementary Table S1). Resting ECG showed a normal electrical axis, sinus rhythm of 69 bpm, and signs of left ventricular hypertrophy. Exercise stress testing revealed no changes in the ST segment or exercise-induced dysrhythmic changes, except for two premature ventricular complexes during the post-exercise period. Hospital admission was recommended for further diagnostic evaluation and assessment of appropriate treatment, including anticoagulants and ICD.

**Figure 2:**
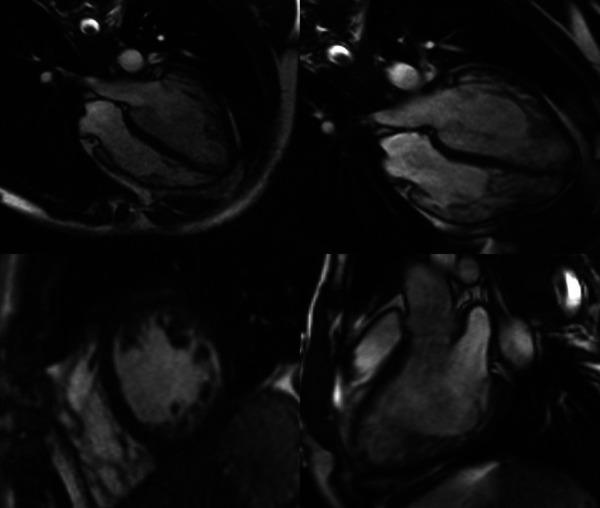
Cine cardiac magnetic resonance images of left ventricular cardiomyopathy with excessive trabeculation. Representative images of cine cardiac magnetic resonance imaging (CMR) steady-state free precession (SSFP) sequences at the time of diagnosis of cardiomyopathy with excessive trabeculation (left panel) and the onset of amiodarone-induced thyroiditis (right panel). The first row of images represents the four-chamber view through the horizontal long axis, whereas the second row consists of the short-axis pre-contrast scan (left panel) and vertical long-axis scan (right panel). Magnetic Resonance Imaging Scanner Siemens Magnetom® Avanto 1.5T (Siemens Medical Solutions, Inc., Hoffman Estates, IL, USA) was used to acquire CMR scans.

On admission, echocardiography and cardiac magnetic resonance imaging were performed to determine the presence of structural deterioration that could be attributed to the observed dysrhythmic changes. Recently performed coronary angiography with no evidence of stenosis or atherosclerosis excluded the ischemic etiology of NSVT. Transthoracic echocardiography using Vivid E95 Cardiac Ultrasound (GE Healthcare, Chicago, IL, USA) revealed a degenerative aortic and mitral valve with normal overture and kinetics maintained, respectively. Myocardial thickness was normal, but global hypokinesia was described while the interventricular septum and the inferoposterior wall of the myocardium showed decreased contractility. The global ejection fraction of the left ventricle was estimated to be 45%. Additionally, transmitral diastolic flow with a biphasic pattern of abnormal LV relaxation (I/IV grade LV diastolic dysfunction) was noted. Cine cardiac magnetic resonance (CMR) steady-state free precession sequences showed a dilated left ventricle with septum hypo-dyskinesia and an ejection fraction of 48%, as well as excessive trabeculation in segments 13, 15, and 16, and a part of segments 7 and 10 with a non-compact to compact myocardium (NC/C) ratio higher than 2.3 in end-diastole (Figure 2). In the late gadolinium enhancement (LGE) sequences with phase-sensitive inversion recovery (PSIR), there were an LGE midwall striae in the basal and midseptums (Supplementary Figure S1).

Pathological non-compaction, distinguished by an NC/C ratio higher than 2.3 in diastole, was the main morphological criterion used for the validation of LVNC two years before the current admission (17). The left ventricular ejection fraction was mildly reduced (LVEF 46%), but the mid-ventricular imbibition of the interventricular septum and its insertion in segment 9 in late post-contrast sequences was minimal then (Supplementary Figure S1). Based on new terminology (16), the patient most likely has dilated cardiomyopathy with excessive trabeculation of the myocardium, according to increased volume of both ventricles and decreased systolic function of the left ventricle with signs of myocardial fibrosis typical for dilated cardiomyopathy (Supplementary Figure S1). Differential diagnosis also included status post myocarditis, but pathognomonic imaging signs of acute myocarditis were not detected at relevant time points (Supplementary Figure S2). The difference in LGE observed between the two CMR imaging time points may suggest disease progression of dilated cardiomyopathy with excessive trabeculation (Supplementary Figure S1) but does not fully explain the observed cECG report.

**Figure 3:**
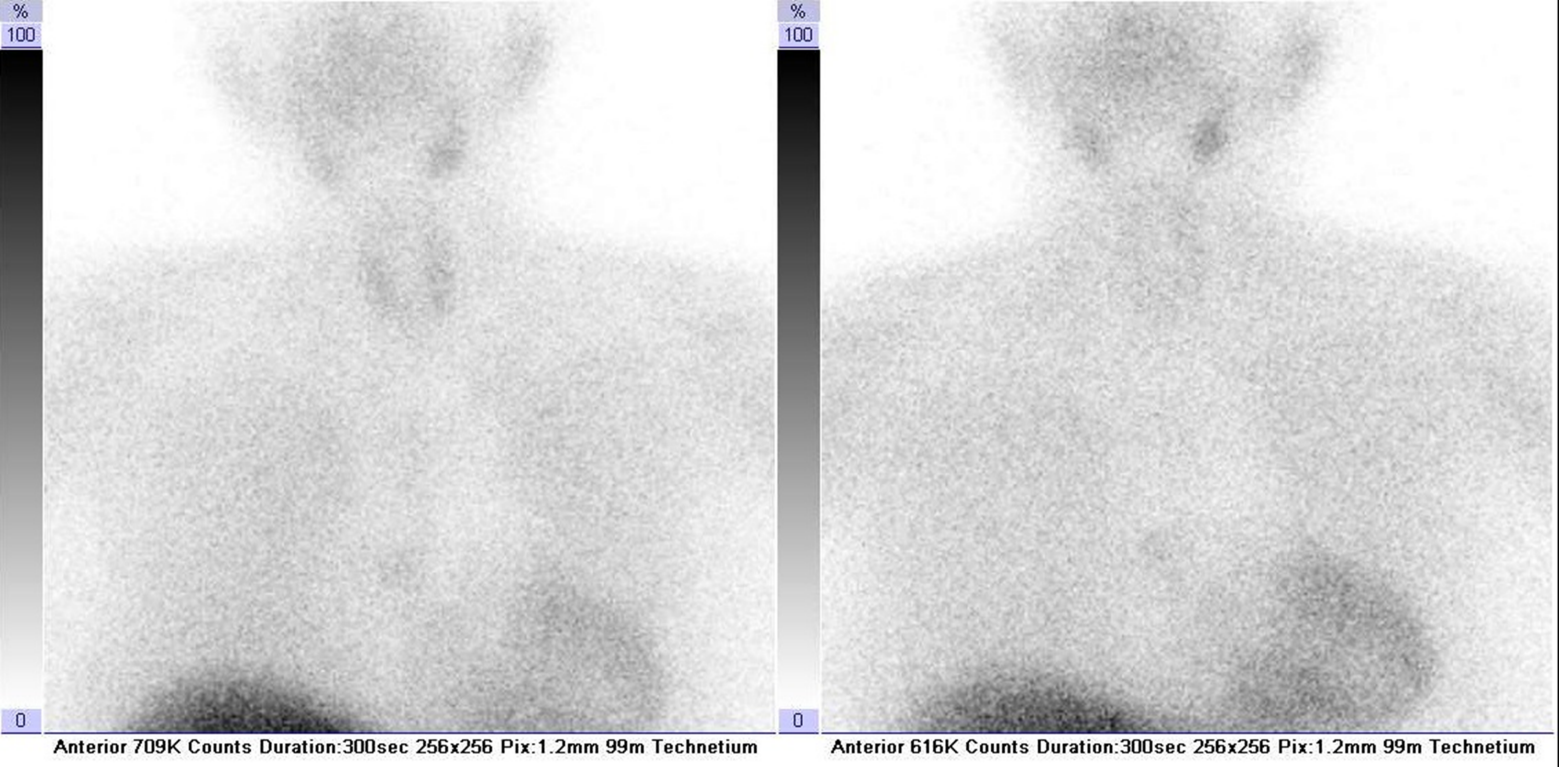
^99m^Tc-sestamibi thyroid scintigraphy confirming the diagnosis of type II amiodarone-induced thyroiditis. Neck and thorax scintigraphy was recorded in the anterior projection at 10 (on the left) and 60 min (on the right) after intravenous administration of 375 MBq of ^99m^Tc-sestamibi. Radiopharmaceutical ^99m^Tc-sestamibi was prepared using a commercially available kit Stamicis® (Curium, Gif-sur-Yvette Cedex, France) and freshly eluted ^99m^Tc pertechnetate (375 MBq; Mallinckrodt, Petten, Netherlands). Symbia T2 dual-head hybrid γ-camera equipped with multipurpose low-energy high-resolution collimators (Siemens Medical Solutions, Inc., Hoffman Estates, IL, USA) was used for recording.

An unmeasurably low thyrotropin level on admission and previous exposure to amiodarone pointed the diagnostic pathway in the direction of the thyroid gland. Nevertheless, the patient's thyroid gland did not appear enlarged or painful on palpation. Suppressed TSH levels were, however, in accordance with reported excessive sweating, reduced appetite, and a 4 kg weight loss over 2 weeks. The next step was to determine free thyroxine levels and thyroid autoantibody titers to investigate the thyroid disorder further. An elevated free thyroxine level of 39.8 pmol/L (reference interval 12–22 pmol/L) and undetectable titers of thyroid-stimulating immunoglobulin, thyroid peroxidase, and thyroglobulin antibodies raised the clinical suspicion of type II AIT in a patient with previous exposure to amiodarone. Neck ultrasound was unremarkable, and color flow Doppler sonography did not show increased thyroid vascularity. Moreover, scintigraphic imaging after intravenous injection of 375 MBq ^99m^Tc-sestamibi showed decreased thyroid uptake (Figure 3). These findings were in accordance with the suggested diagnosis, and prednisone was introduced to treatment. Additionally, carvedilol treatment was changed to a combination of propranolol 20 mg and ramipril 2.5 mg. Due to a concomitant paroxysm of atrial fibrillation detected during the hospital stay, dabigatran 150 mg twice daily was administered. Regular follow-up of thyroid hormone levels was performed during the hospital stay.

**Figure 4:**
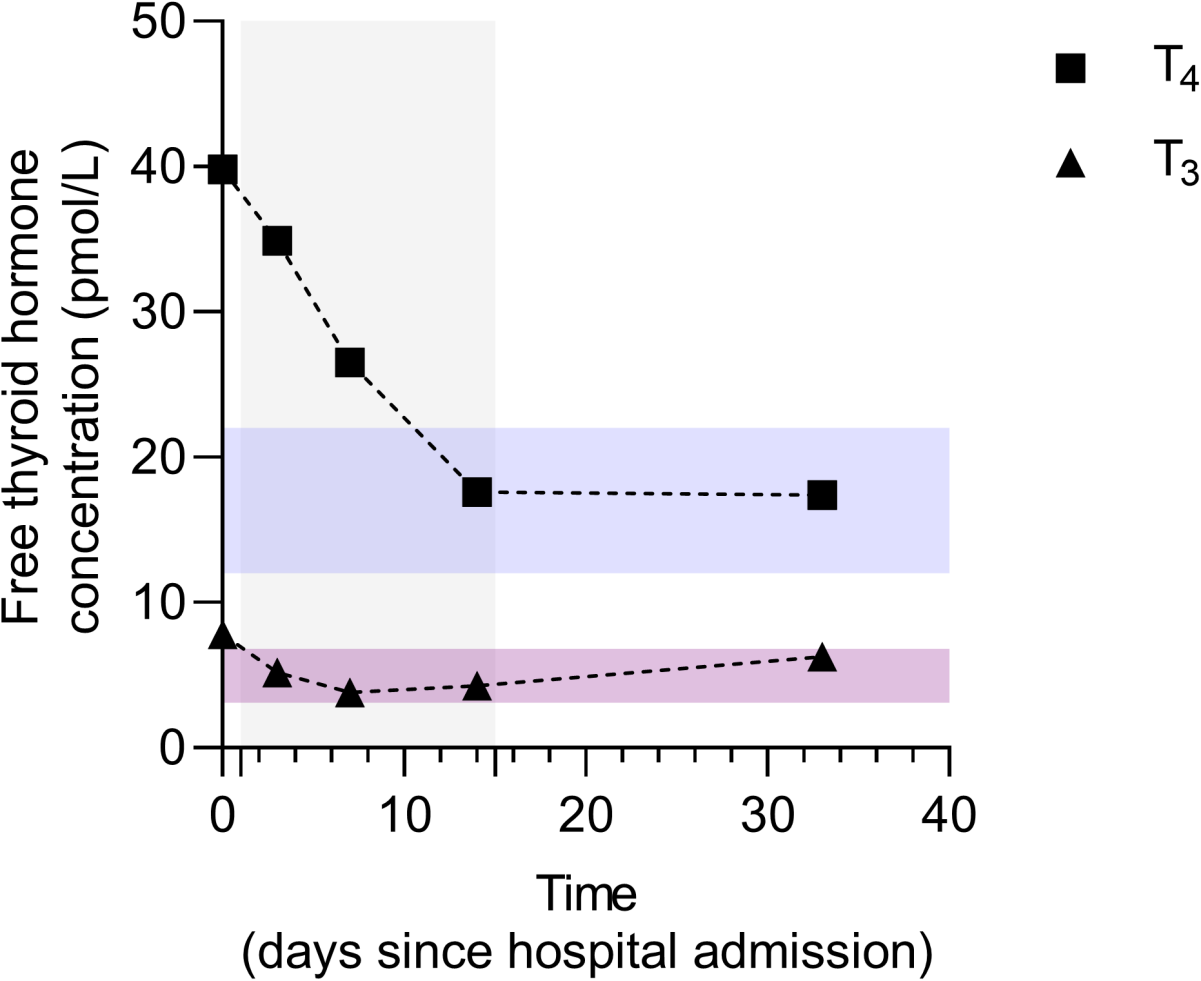
Dynamics of free thyroid hormone concentrations before, during, and after immunomodulatory treatment of amiodarone-induced thyroiditis. Data are presented as individual values of free thyroxine (FT_4_) and triiodothyronine (FT_3_) concentrations before, during, and after prednisone treatment. The treatment duration is marked as a grey background area ranging from day 1 to day 15. Reference intervals for FT_4_ and FT_3_ levels are indicated by blue and purple transparent areas, respectively. Serum concentrations of FT_4_ and FT_3_ were determined by electrochemiluminescence immunoassay (ECLIA) using commercially available assays Elecsys FT3 III (Roche Diagnostics GmBH, Manheim, Germany) and Elecsys FT4 IV (Roche Diagnostics GmBH, Manheim, Germany) on the Cobas® 8000 analyzer series cobas® e 801 analytical unit (Roche Diagnostics International, Ltd, Rotkreuz, Switzerland).

Patient-reported adherence to treatment was adequate and no adverse or unanticipated events were reported. After only a week of corticosteroid treatment, TSH was 0.01 mIU/L and free thyroxine level dropped to 26 pmol/L (Figure 4). After 2 weeks, the target levels of thyroid hormones were reached, and prednisone treatment was gradually de-escalated until complete discontinuation (Figure 4). Thirty-three days after hospital admission due to NSVT, the cECG determined ventricular heterotopy of 57 solitary, four pairs, and one triplet of ventricular extrasystoles (Supplementary Table S1). Additionally, only 8 solitary supraventricular extrasystoles have been reported (Supplementary Table S1). At the same time point, follow-up TSH was 6.28 mIU/L (reference interval 0.27–4.20 mIU/L) and free thyroxine was 17.38 pmol/L. Clear improvements in both biochemical and electrocardiographic parameters were observed after immunomodulatory treatment of type II AIT in a patient with cardiomyopathy and excessive trabeculation. Nevertheless, regular endocrinological and cardiological follow-ups are warranted. Finally, patient-reported outcomes at the 10-month follow-up were consistent with the biochemical and electrocardiographic remission after the reported AIT episode.

### 2.1. Patient perspective

“When I got admitted to the hospital, I was afraid. I did not know what caused that feeling of tiredness, but I was relieved once the doctors found the correct diagnosis. They explained everything to me, and I was very satisfied with the care during the hospital stay. Now, I am living a bit more cautiously, and I know my limitations. I understand how important it is to take my medications every day and attend regular follow-ups. At this point, I do not have any fears but know where to seek help if it changes. I would be happy to help other doctors learn from my case.”

## 3. Discussion

Excessive trabeculation of the left ventricle is a rare ventricular phenotype, affecting between 0.05% and 0.25% of the population, routinely quantified by cardiac imaging techniques, including CMR and echocardiography (18). The abnormal maturation process during embryogenesis is a well-established underlying mechanism for the increased number and prominence of endomyocardial trabeculations in neonates and children (18). In contrast, the pathogenesis of excessive trabeculation in adults has recently been recognized as a normal variation or cardiac adaptation to conditions with increased preload (16). Controversies remain regarding adults with the combination of cardiomyopathy and excessive trabeculation. Although current evidence suggests that the extent of trabeculation in adults with left ventricular cardiomyopathy does not influence the prognosis or management of these patients, large multicenter studies on long-term outcomes supporting specific clinical decisions are still lacking (16). According to previous reports, symptomatic patients with left ventricular cardiomyopathy with excessive trabeculation (obsolete terms: left ventricular non-compaction cardiomyopathy or spongiform cardiomyopathy) may present throughout life with progressive systolic dysfunction of the left ventricle and carry an increased risk of malignant arrhythmias and thromboembolic events independent of the coexisting trabeculation (16). Adult patients with confirmed cardiomyopathy, regardless of excessive trabeculation, should therefore be closely monitored and multimodally treated for congestive heart failure, hypercoagulable state, and cardiac rhythm abnormalities to prevent life-threatening arrhythmias with medications and/or ICD and to reduce cardiovascular mortality (19, 20). Unfortunately, the choice of antiarrhythmic medications in patients with dilated cardiomyopathy and reduced systolic function is limited to beta-adrenoceptor-blocking agents and amiodarone.

The potent antiarrhythmic action of amiodarone tends to outweigh the risks of its toxicity, which was first described more than half a century ago (21, 22). Amiodarone contains two iodine atoms, which account for more than one-third of its molecular weight. In patients with an underlying autoimmune disease, the iodine-mediated effect of amiodarone is a result of a critical increase in intrathyroidal iodine concentrations, causing failure to escape from the Wolff-Chaikoff effect, described as a reduction in thyroid hormone levels after exposure to a large amount of iodine, or the Jod-Basedow effect, characterized by excessive thyroid hormone synthesis in the autonomous regions of the gland (21). Nevertheless, amiodarone exerts an iodine-independent intrinsic effect, including inhibition of outer ring 5′-monodeiodination of thyroxine, blockade of triiodothyronine receptor binding to nuclear receptors, subsequent expression of thyroid hormone-related genes, and a direct cytotoxic effect on thyroid follicular cells (22). The crucial difference in the underlying pathogenesis requires a cautious approach to the diagnosis and treatment of patients with life-threatening AIT.

The challenging path to the correct diagnosis and treatment of patients with AIT has been extensively studied and described in the literature (3–8). However, very few studies have shown a therapeutic approach to patients with underlying cardiac disorders and type II AIT (10–13). Similar to our case, a young patient with dilated cardiomyopathy and type II AIT mimicking low cardiac output syndrome presenting with general malaise and nausea was effectively treated with prednisolone (10). In other reports, patients did not respond to oral glucocorticoid treatment and required definitive surgical treatment of the hyperactive thyroid gland (11–13). The decision to treat the thyroid disorder before considering radiofrequency ablation or, in our case, ICD implantation is similar to the case reported by Pillarisetti et al. (14). Notably, hyperthyroid conditions regardless of their etiology may cause or aggravate heart rhythm disorders (23), whereas adequate treatment of hyperthyroidism reduces the number of complex arrhythmias. Here, we report a case of a patient with arrhythmia of complex origin, including the synergistic arrhythmic effect of amiodarone-induced thyroiditis and dilated cardiomyopathy as the underlying disorder. Any delay in the treatment of thyroiditis would cause the tachyarrhythmia to last longer and, therefore, not only increase the risk of sudden death but also lead to further dilation of the left ventricle and subsequent decline in the systolic function due to elements of tachycardiomyopathy. Early diagnosis and correct treatment in our unique case of type II AIT in a patient with a rare diagnosis of left ventricular cardiomyopathy with excessive trabeculation showed a significant improvement in biochemical and echocardiographic parameters. These reported cases imply the necessity of a multidisciplinary and individualized approach to every patient with type II AIT and underlying cardiac disease.

Treatment of reversible causes of cardiac rhythm abnormalities such as type II AIT should be considered before choosing other treatment modalities, particularly in patients with structural cardiac disorders. The importance of a multidisciplinary approach in complex cases such as the one reported thus cannot be emphasized enough.

## Data Availability

The original contributions presented in the study are included in the article/**Supplementary Material**, further inquiries can be directed to the corresponding author.
