# Supplementary material for: When amiodarone-induced thyroiditis meets cardiomyopathy with excessive trabeculation: a case report
Source: Front Cardiovasc Med. 2023 Jul 21;10:1212965. doi: 10.3389/fcvm.2023.1212965 (PMC10401478; doi:10.3389/fcvm.2023.1212965)
Supplement: Supplementary file 1 [file Datasheet1.pdf]

Supplementary Material

When amiodarone-induced thyroiditis meets cardiomyopathy with excessive trabeculation: a case report

Dora Gašparini*, Damir Raljević, Vesna Pehar-Pejčinović, Tihana Klarica Gembić, Viktor Peršić, Tamara Turk Wensveen

*** Correspondence:** Dora Gašparini, dora.gasparini@uniri.hr

1 Supplementary Data

1.1 Supplementary Figures

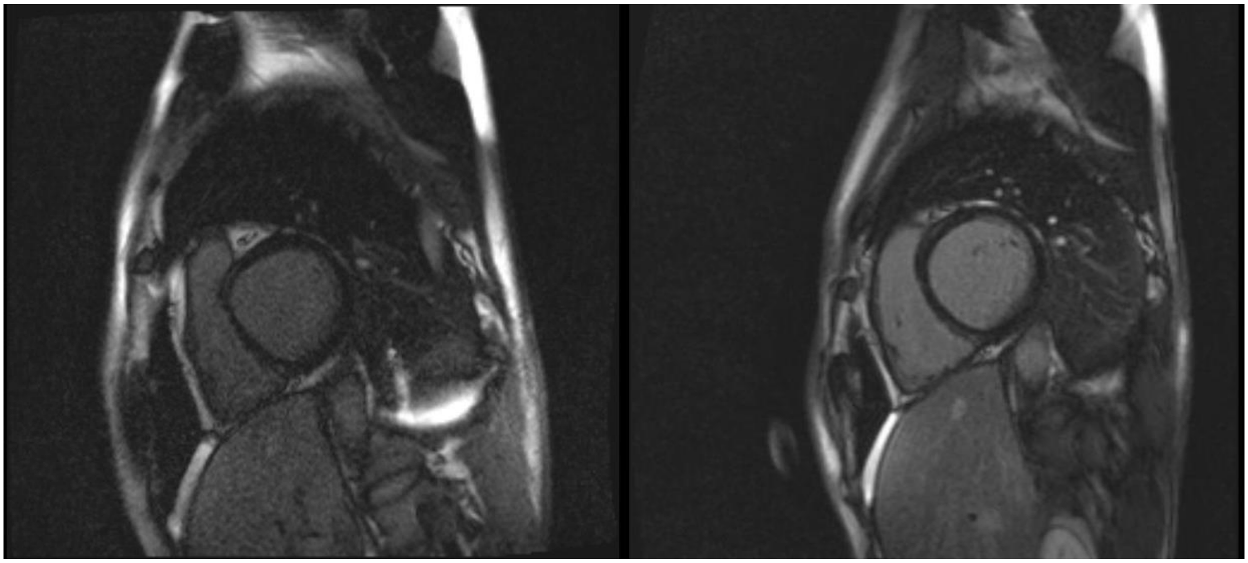

Supplementary Figure 1. Late gadolinium enhancement imaging scans with regional distribution of contrast typical for dilated cardiomyopathy. Cardiac magnetic resonance (CMR) with late gadolinium enhancement (LGE) short-axis scans with phase-sensitive inversion recovery (PSIR) representing regional distribution of LGE typical for dilated cardiomyopathy or status post myocarditis at the time of diagnosis (left panel) and a minimal increase in LGE at the onset of amiodarone-induced thyroiditis (right panel). Magnetic Resonance Imaging Scanner Siemens Magnetom® Avanto 1.5T (Siemens Medical Solutions, Inc., Hoffman Estates, IL, USA) was used to acquire CMR scans.

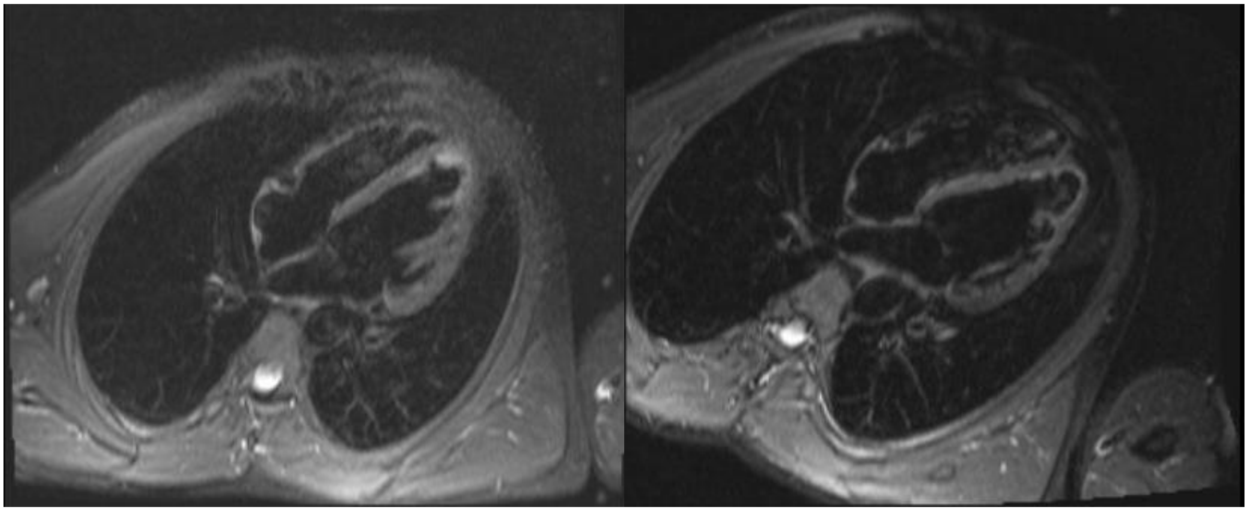

Supplementary Figure 2. Exclusion of acute myocarditis by T2-weighted images. Representative T2-weighted images of cardiac magnetic resonance (CMR) at the time of diagnosis (left panel) and at the onset of amiodarone-induced thyroiditis (right panel) lacking signal hyperintesities, a typical finding for acute myocarditis.

1.2 Supplementary Tables

Supplementary Table 1. Continuous electrocardiographic monitoring reports before and after immunomodulatory treatment of amiodarone-induced thyroiditis. bpm=beats per minute; HR=heart rate; NA=not applicable; SVE=supraventricular extrasystole; VE=ventricular extrasystole.

	Before treatment	*After treatment*
VE Total	882	57
V-Pair Total	80	4
V-Run Total (Total Beats)	114 (412)	1 (3)
Longest V-Run, HR in bpm	36, 102	3, 52
Maximum HR V-Run, HR in bpm	3, 188	3, 52
VEs per 1000/per Hour	8.55/37.37	0.76/2.70
SVE Total	1715	8
SVE-Pair Total	0	0
SV-Run Total (Total Beats)	10 (1708)	0
Longest SV-Run, HR in bpm	1387, 129	NA
Maximum HR SV-Run, HR in bpm	1387, 129	NA
SVEs per 1000/per Hour	16.62/72.67	0.11/0.38
